# A prediction‐based test for multiple endpoints

**DOI:** 10.1002/sim.8724

**Published:** 2020-09-15

**Authors:** Robert N. Montgomery, Jonathan D. Mahnken

**Affiliations:** ^1^ Department of Biostatistics & Data Science University of Kansas Medical Center Kansas City Kansas USA

**Keywords:** correlated endpoints, multiplicity, O'Brien's OLS

## Abstract

This article introduces a global hypothesis test intended for studies with multiple endpoints. Our test makes use of a priori predictions about the direction of the result of each endpoint and we weight these predictions using the sample correlation matrix. The global alternative hypothesis concerns a parameter, ϕ, defined as the researcher's ability to correctly predict the direction of each measure, essentially a binomial parameter. This allows for the test to include expected effects that are all positive, all negative or both while still using the cumulative information across those endpoints. A rejection of the null hypothesis ($H_{0}:\phi \leq \phi_{0}$) provides evidence that the researcher's underlying theory about the natural process provides a better prediction of the observed results relative to the null hypothesized predictive ability, thus indicating the theory is worthy of further study. We compare our test to O'Brien's ordinary least squares (OLS) test and show that for small samples and situations where the effect is not in the same direction across all endpoints our approach has better power, while if the effect is equidirectional across all endpoints the OLS test can have greater power.

## 1 INTRODUCTION

In contemporary biomedical science many studies involve multiple outcomes of interest. While significant advances in the methodologies to deal with multiplicity have been developed, the tendency in biomedical research to measure as many endpoints of interest as possible within each study is not well suited to balancing sufficient multiplicity adjustments along with sample size, financial, and logistic constraints, especially in early stage research where sample sizes are typically small. We present a paradigm for experimental testing that controls the type I error rate of the overall experiment (or more specifically of the global research hypothesis) while maintaining adequate power. We believe this methodology will be especially useful for settings where a large number of endpoints are of interest, with no “primary” endpoint and the sample size is relatively small. In addition our test is not restricted to situations where a common effect size, or direction, is expected across all endpoints. This allows for cumulative information from endpoints where an “improvement” might be in opposite directions to be incorporated in a single test.

The scenarios in which multiple endpoints arise are varied, ranging from trials with distinct outcomes of interest, such as endpoints for both efficacy and safety, to trials where the complex nature of or the inability to directly measure the hypothesis of interest necessitates multiple endpoints that can be used as proxies.^1^ Having more than one endpoint can have serious implications on the operating characteristics of a study and typically needs to be addressed. This has led to a wealth of literature about the topic,^2^ including guidance from government agencies about best practices.^1^, ^3^ However, despite widespread knowledge of the need for adequate multiplicity corrections when faced with multiple endpoints a common default continues to be the designation of a primary endpoint, with all others analyzed as secondary, or exploratory, even if there is not a true “primary” endpoint. Our test provides a method for arriving at a decision when there is not a true primary endpoint, a common occurrence in early stage research when the validity of the overall theory or research hypothesis is being tested. We incorporate the correlation matrix of the measured endpoints along with a set of researcher's predictions to form our test statistic.

### 1.1 Review of similar methodologies

Typical approaches for addressing multiplicity include simple single step procedures, such as the Bonferroni or Sidak adjustment,^4^ multistep procedures such as Holm's method^5^ as well as global procedures such as Hotelling's *T*^2^. However, the most comparable existing methodology to our proposed test are the global tests developed by O'Brien.^6^

O'Brien proposed three tests: an ordinary least squares (OLS) test, a generalized least squares (GLS) test, and a nonparametric rank sum (RS) test. Part of the motivation for the development of the tests stemmed from the desire to not only determine a difference between groups across endpoints, but also to perform well in situations where “improvement was demonstrated consistently among the various endpoints,”^6^ that is, if the effect across the endpoints was assumed to be in the same direction and of similar magnitude. O'Brien's test has been shown to outperform other approaches such as Hotelling's *T*^2^ test when this assumption is true.^6^, ^7^ The global null hypotheses for O'Brien's set of tests is

$$H_{0}:\delta =0$$

where δ denotes the vector of mean differences, $\delta_{k}$ of interest between two groups for the *k*th endpoint of interest. By making the assumptions that the effect size for each endpoint, $\delta_{k}/\sigma_{k}$, was equal to some constant, λ then O'Brien reformed the null hypothesis to be

$$H_{0}:\lambda =0$$

against the alternative that λ>0.^8^ The OLS and GLS tests are sums of t‐test statistics for each individual endpoint. The difference between the two tests is that the GLS statistic weights the different endpoints depending on the sample correlation matrix, such that all weights are not required to be equal.^9^ This allows for more weight to be given to endpoints that are less associated with other endpoints. Both tests have been shown to outperform methods that do not take knowledge of directional alternatives into account and comparisons of the OLS and GLS tests have been made. However, direct comparisons between the OLS and GLS test statistics are difficult due to the fact that the GLS has been shown to be very liberal under various types of correlation matrices and regardless of the approximation used for the degrees of freedom of the test statistic.^10^ Thus, direct power comparisons between the two are not meaningful. In addition, while the GLS test makes use of the sample correlation matrix to weight endpoints this is done through the row sums of the inverse of the sample correlation matrix which can lead to negative weights in certain situations, an undesirable property. Thus, the OLS test is typically recommended^8^ and will be the main comparison for our proposed method. Both test statistics have asymptotic F distributions with several different proposed estimators of the degrees of freedom. The OLS and GLS tests do not have an exact distribution, and the small sample approximations can be poor,^10^ in these situations the RS test could be used. Our proposed test is similar to the OLS and GLS test statistics, notably our method also uses the sample correlation matrix to weight different endpoints and the settings in which either method could be used are similar. In Section 6, we examine the relative merits of the two tests under various scenarios.

### 1.2 Motivating example

Consider an experiment in which measures of arterial spin labeling (ASL), a measure of cerebral blood flow, were collected in different regions of the brain. This data was collected as part of a larger study examining the relationship between exercise and Alzheimer's disease that measured pre‐post changes after a 12 week exercise intervention in 11 older adults. The dataset consists of ASL measures on six regions of the brain: BA46, Frontal mid, Hippocampus, M1, Superior Parietal and Precuneus. The research hypothesis was that there would be structural changes in the brain following the intervention and the changes in ASL function as a proxy for this more general hypothesis. No single region was considered to be of primary interest, therefore we needed to come to a global conclusion about whether there were structural changes in the brain. In addition the primary investigator believed that ASL values would increase in each individual region and that this would provide evidence for an improvement postintervention. We wanted to test whether the PI's theory was correct, that this intervention would lead to increases in ASL values. We also wanted to take into account the predicted direction of the pre‐post change, that of an increase, and that the expected increases might be small, that is, not “statistically significant” in a set of univariate analyses. It was expected that there would be fairly high correlation between the ASL measures on the various endpoints due to the measurements being of blood flow within the same individual. In addition, the sample size was relatively small (n = 11). This type of setting, a small n relative to the number of endpoints of interest, along with directional predictions of the outcome of the endpoints that might be individually small and correlated motivated the development of our test statistic. We note that while in this example all predicted effects are in the same direction our method can incorporate predicted effects in different direction, e.g. some expected to increase and some decrease.

In Section 2, we introduce the test statistic and associated hypothesis test, Section 3 presents an empirical assessment of the normal approximation, in Section 4 we look at the sensitivity of using a sample correlation matrix for both the normal approximation and the exact test, Section 5 discusses the choice of the parameter ϕ, Section 6 compares our method to O'Brien's test and applies both tests to the ASL data and finally in Section 7 we discuss the methodology presented including its limitations and areas for future work. The appendix contains a proof showing the Lindeberg condition of the central limit theorem holds and the supplementary information provides R code for the implementation of the proposed test.

## 2 TEST STATISTIC AND ASSOCIATED TEST

Let n represent the number of experimental units, and *m* be the number of endpoints measured on each experimental unit. For each endpoint the researcher makes a prediction about the direction, for example, with a pre‐post study the prediction for the first endpoint, *m*_1_, could be an increase, and for *m*_2_ a decrease. We set our predictions to be one sided, so that without loss of generality we describe these as being predictions of either a positive or negative result for each endpoint while not restricting the predictions to be all positive or all negative. Let **p** be an *m* × 1 vector of the results of the predictions for the endpoints, where *p*_*i*_, the *i*th value of the vector, is an indicator function that equals 1 if the prediction on endpoint *i* is correct, and 0 if the prediction is incorrect based on the observed sample. Let **C** represent an *m* × *m* correlation matrix between the endpoints, where $\rho_{ij}$ is the pairwise correlation between endpoint *i* and *j*, which we estimate with the sample correlation *r*_*ij*_. Any type of correlation measure can be used with the choice depending on the underlying data,^11^ for all examples and simulations we use Pearson's correlation coefficient.

For the *i*th measure we have defined a weight *w*_*i*_, *i* = 1, … , *m*, that is, the inverse of the sum of the squared pairwise correlations for the *i*th row of **C**, that is

$$w_{i}={\left(\overset{m}{\underset{j=1}{\sum }}r_{i,j}^{2}\right)}^{-1}$$

The weight *w*_*i*_ will take a value of 1/*m* for all *i* when there is a perfect pairwise association (positive or negative) between each measure and will take a value of 1 for all *i* when the endpoints are independent. Thus W, the sum of these weights, will equal 1 if the correlation matrix is a matrix of ones and will equal *m* if the correlation matrix is an identity matrix. Conceptually we view W as an estimate of the number of “unique” or effective endpoints being considered, similar in spirit to the idea of the effective number of variables advanced by several authors in significance threshold correction.^12^, ^13^ It's also similar the weight used in O'Brien's GLS test which uses the inverse of the sum of the pairwise correlations with the same goal of weighting more independent variables more heavily. However, since the weights for the GLS test do not square the elements of the sample correlation matrix the weights can be negative, leading to the possibility of rejecting the null hypothesis in favor of the alternative that λ>0 when in fact the effect is in the opposite direction on every endpoint.^8^

For our proposed test when the endpoints are perfectly independent *W* = *m* implying we have *m* unique endpoints, while if the endpoints are perfectly dependent *W* = 1 implying there is effectively only one independent endpoint. For levels of correlation between these extremes 1 < *W* < *m* indicating that some of the endpoints measure similar attributes.

Our test statistic increases in value for each correct prediction by the corresponding weight *w*_*i*_. We define our test statistic as

$$T_{m}=\overset{m}{\underset{i=1}{\sum }}p_{i}w_{i}$$

where 0 ≤ *T*_*m*_ ≤ *W*. Notably, larger values of *T*_*m*_ indicate experimental results more aligned with the researcher's hypothesized predictions, while those closer to zero imply less concordance between prior predictions and experimental results. It's important to note that for the same number of correct predictions the value of the test statistic will change depending on which endpoints were correctly predicted, thus predicting measures with higher weights, will result in larger test statistic values. We give greater importance to correctly predicting endpoints that are more independent of the other variables in the dataset, in this way correctly predicting a large amount of highly correlated measures may lead to a relatively small test statistic.

### 2.1 Test statistic under the null

Our null hypothesis is that the researcher's predictive ability ϕ is less than or equal to $\phi_{0}$, that is, $H_{0}:\phi \leq \phi_{0}$. The parameter ϕ is chosen in regard to the specific experiment of interest. For example, in early discovery experiments if the researcher was able to predict the direction of more than $\phi_{0}=0.50$, that is, 50%, of the endpoints then perhaps that would be enough to warrant further study because it would indicate that the researcher's theory was able to predict what would happen at a rate better than chance. Under our null hypothesis we assume that the results of each prediction *p*_*i*_ follow a Bernoulli distribution with success parameter ϕ. We also assume that the weights, *w*_*i*_, are independent of the predictions. If the weights are treated as fixed and the predictive ability on all endpoints is equal our test statistic is therefore a weighted sum of Bernoulli random variables, with expected value $E[T_{m}]=\phi \sum_{i=1}^{m}w_{i}$ and $\mathrm{Var}(T_{m})=\phi \cdot (1-\phi )\sum_{i=1}^{m}w_{i}^{2}$.

If the correlation matrix **C** is such that the off‐diagonal values are not all equal then there are a discrete number of unique $w_{i}^{\prime }s$ and the vector **p** can take on 2^*m*^ permutations, as each *p*_*i*_ ∈ {0,1}. Given a sample correlation matrix we can calculate the exact distribution of the test statistic. In doing this we need to consider that different combinations of correct predictions will result in different values of the test statistic even if the overall number of correct predictions is the same. Unlike a binomial probability we do care about which specific combination leads to *x* correct predictions, thus the pmf of our test statistic can be considered as a binomial pmf without the constant mx where $x=\sum_{i=1}^{m}p_{i}$, the number of correct predictions (ie, 1's) in the prediction vector **p**.

More formally the pmf is

$$f(T=p^{T}w)=\phi_{0}^{\sum p_{i}}{\left(1-\phi_{0}\right)}^{m-\sum p_{i}}$$

The sum over all 2^*m*^ permutations of **p** (and thus **p**^*T*^**w**) equals 1 as would be expected. As an example, with *m* = 2 and $\phi_{0}=0.50$ the probability of correctly predicting 1 of the endpoints is 0.50^1^(1 − 0.50)^1^ = 0.50^2^ = 0.03125, of course there are 21=2 sets of correct predictions which would result in two different tests statistics depending on the weight. Under the null hypothesis these two values of the statistic have the same probability. When $\phi_{0}=0.50$ the probability of all possible values of *T*_*m*_ will be equal due to the symmetry of the binomial; however, as $\phi_{0}$ deviates from 0.50 the probability of observing different values of the test statistic will change.

### 2.2 Special cases

There are two special cases concerning the distribution of our test statistic, when **C** = ***J***_*m*_, an *m* × *m* matrix of ones, and when **C** = **I**, the identity matrix.

For **C** = **I**, *w*_*i*_ = 1 for all *i* thus our test statistic can be written as:

$$T_{m}=1\cdot \overset{m}{\underset{i=1}{\sum }}p_{i}$$

The sum of independent Bernoulli random variables is a Binomial random variable. Thus, the test statistic would simply follow a Binomial(m, $\phi_{0}$).

If the sample correlation matrix **C** = ***J***_*m*_ (with the off diagonals being either positive or negative one) then *w*_*i*_ = 1/*m* for all *i*. Our test statistic in this scenario can be written as:

$$T_{m}=\frac{1}{m}\overset{m}{\underset{i=1}{\sum }}p_{i}$$

If we let $X=\sum p_{i}$, and let Y be the transformation $Y=\frac{1}{m}X$ we can show the probability mass function of *T*_*m*_ is:

my·mpy·m(1−p)m−m·y

with support *y* = {0, 1/*m*, 2/*m*, … ,1}. Thus, *T*_*m*_ is simply a linear transformation of a Binomial random variable in the completely dependent case. For sample data the only realistic way either of these scenarios could occur would be through error, or in a contrived way such as measuring the same variable but with different units, for example, height in inches, centimeters and meters.

### 2.3 Decision rule

We are most concerned with whether the researcher's theory provides an advantage in understanding the outcome of different endpoints, thus we define our null and alternative hypotheses as follows:

$$\begin{array}{ll} H_{0}:\phi \leq \phi_{0} & \\ H_{1}:\phi >\phi_{0}. & \end{array}$$

We also suggest that *T*_*m*_ ≥ 1 in order to reject the null, that is, we require the sum of correct scores equal at least 1. This forces the researcher to correctly predict every endpoint when the endpoints are perfectly dependent, thus simply correctly predicting linear, or monotonic combinations of other endpoints provides no advantage. We consider the sum of *T*_*m*_ to be the number of effective endpoints correctly predicted and thus it is intuitive we would require the researcher to correctly predict endpoints with a value of at least one. In situations where the Type I error rate was of most concern ϕ would be close to 1, and in situations where power was the main concern ϕ would be closer to 0, although we would recommend $\phi_{0}\geq 0.5$ without strong reasoning otherwise.

## 3 NORMAL APPROXIMATION

The exact distribution for *T*_*m*_ becomes computationally difficult to calculate as the number of endpoints increases, with *m* = 30 the number of possible permutations of **p**, the prediction vector, is over 1 billion. In Appendix B we have shown that the sum of the $T_{i}^{\prime }s$ satisfies the Lindeberg CLT, indicating that as the number of endpoints increase the central limit theorem will apply as long as the value of $\phi_{0}$ is bounded away from 0 and 1.^14^ We show these restrictions on $\phi_{0}$ will be met as we let *m* → *∞*. We also conducted a simulation study for various values of ϕ and *m*. We found that if *m* is large enough and ϕ is not too close to either boundary we can approximate our test statistic with the following normal distribution:

$$T_{m}∼\text{Normal}(\mu ,\sigma )$$

where μ=ϕ·W and $\sigma =\sqrt{\phi (1-\phi )\cdot \sum w_{i}^{2}}$.

**FIGURE 1:**
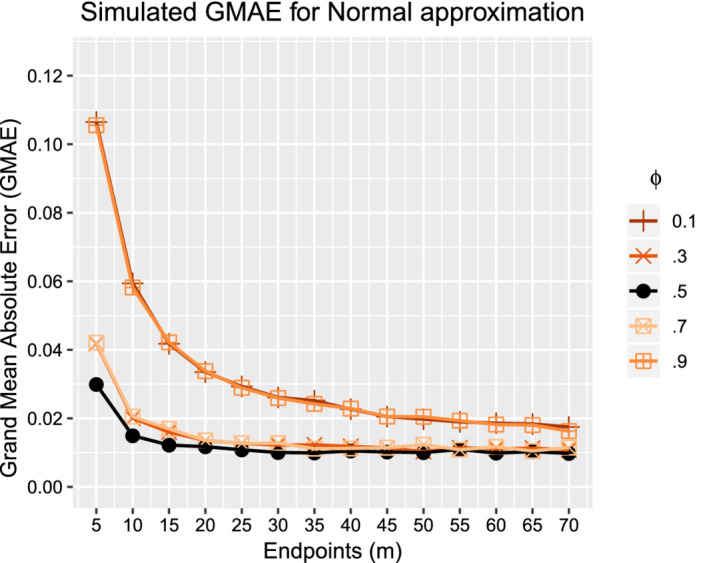
For each combination of *m* and $\phi_{0}$ we calculated the GMAE. We see a clear decrease in GMAE as the number of endpoints increases. GMAE, grand mean absolute error [Colour figure can be viewed at wileyonlinelibrary.com]

We looked at values of *m* ranging from 20 to 70 in increments of 5 due to the fact that with *m* > 20 the calculation of the exact distribution becomes computationally expensive. We generated random correlation matrices using the R^15^ package clusterGeneration^16^ which uses partial correlations and a recursive method to generate a random *m*‐dimensional covariance matrix which we converted to a correlation matrix.^17^ The method depends on the dimension of the correlation matrix and on a parameter $\alpha_{d}$. We used $\alpha_{d}=1$ which is a special case that is uniform over the space of positive definite correlation matrices. For each value of *m*, we simulated 100 different correlation matrices, for each of these correlation matrices we randomly generated 1000 sets of predictions from a Bernoulli($\phi_{0}$) distribution. We used these predictions and the correlation matrix to estimate the exact CDF of *T*_*m*_ which we then compared with the approximate CDF using the normal approximation. We calculated the mean absolute error (MAE), that is, the sum of absolute differences between the values divided by the number of approximated percentiles, 1000 in this case. We took the mean of these 100 000 MAE's (1000 for each of the 100 correlation matrices) to form the grand mean absolute error (GMAE), averaging over the generated correlations matrices for each combination of *m* and ϕ. Figure 1 shows the results.

When $\phi_{0}$ is near one of the boundaries the approximation is poor especially with a small number of endpoints. However, as the number of endpoints increases the GMAE decreases even when $\phi_{0}$ is near the boundary. With a more moderate value of $\phi_{0}$ the normal approximation is much closer to the value from the exact distribution, with $\phi_{0}=0.50$ the error quickly approaches 0.01 on average.

## 4 SENSITIVITY ANALYSIS OF SAMPLE CORRELATION MATRIX

A concern when applying our method could be the instability of the sample pairwise correlations for small sample sizes. Some estimates of the necessary sample size for stable estimates of correlation are in the hundreds depending on the application.^18^ Most of the literature concerning adequate sample sizes for sample correlations has been focused on doing hypothesis testing on a single sample correlation or estimating a confidence interval for a single sample correlation. While we are not directly interested in using the pairwise correlation for testing it is of concern that unstable pairwise correlations could affect our weights and therefore affect the overall test statistic. To examine this situation, we conducted a simulation study. We let ϕ=(0.30,0.40,0.50,0.60,0.70,0.80,0.90) for $\phi_{0}=0.50$ and ϕ=(0.50,0.60,0.70,0.75,0.80,0.85,0.90) for $\phi_{0}=0.70$. We generated a “true” correlation matrix, then sampled from a multivariate normal in R using the true correlation matrix to generate a sample, calculated the “true” weights and the sampled weights and simulated our tests for n = 20. We kept track of the proportion of times that our test came to the same conclusion, these values are presented in Table 1. For *m* < 20 we used the exact test while for *m* ≥ 20 we used the normal approximation.

**TABLE 1:** Proportion of tests coming to the same conclusion for combinations of n, $\phi_{0}$ and *m*

n	$\phi_{0}$	*m* = 5	*m* = 10	*m* = 15	*m* = 20	*m* = 25	*m* = 30	*m* = 35	*m* = 40	Average agreement
10	0.50	100	97.1	96.6	97.4	97.7	97.9	98.1	98.2	**97.6%**
	0.70	100	97.0	96.4	98.0	97.0	96.3	96.2	96.9	**96.8%**
20	0.50	100	97.9	97.4	97.9	98.1	98.4	98.5	98.4	**98.1%**
	0.70	100	97.8	97.0	98.5	97.8	96.8	96.6	97.2	**97.4%**

*Note*: Average agreement calculations do not include *m* = 5 for $\phi_{0}=0.7$ since the null cannot be rejected in this case.

The results show that estimating the true correlation matrix with a sample correlation matrix, even for with a relatively small n, leads to the same conclusions as if we had the true correlation matrix. This holds for both the exact test statistic and the normal approximation. Despite these results if there is concern over the validity of the sample correlation matrix, we would suggest eliciting expert opinions or using historical studies to estimate the correlation matrix, which can then be used for a sensitivity analysis.

## 5 CHOICE OF ϕ

The choice of the hypothesized value $\phi_{0}$ is a critical decision that needs to be made before the data is analyzed. A higher choice of $\phi_{0}$ will lead to a decrease in power. If the normal approximation will be utilized the accuracy of the approximation is maximized for $\phi_{0}=0.50$. It's important to note that for some combinations of *m* and $\phi_{0}$ it is impossible to reject the null hypothesize at a given α level due to discreteness. Table 2 provides the minimum *m* that can be chosen for a given $\phi_{0}$ such that the null hypothesis could still be rejected at α=0.05. Note that for these combinations of $\phi_{0}$ and *m* a correct prediction would need to be made on every endpoint to reject the null.

**TABLE 2:** Minimum required *m* for hypothesized value $\phi_{0}$ with α=0.05

$\phi_{0}$	0.50	0.60	0.70	0.80	0.90
Minimum required *m*	5	6	9	13	29

## 6 PERFORMANCE

### 6.1 Simulated results

To assess the performance of our proposed test we have estimated the power and Type I error control under various simulation settings. In addition we have compared our proposed test to O'Brien's OLS test. A direct comparison of the two tests is difficult due to the two tests having different alternative hypothesis (OLS: $H_{1}:\lambda >0$, Proposed: $H_{1}:\phi >\phi_{0}$). The OLS test considers the global alternative of an increase (or decrease) on every endpoint between groups. Essentially the alternative is that the researcher simply predicts which direction (up or down) they think all endpoints will go. This does restrict the application of the OLS test to only include endpoints that are expected to change in the same direction. Our proposed test considers a null concerning the researcher's ability to predict the direction of differences between groups on each endpoint, typically set at $\phi_{0}=0.50$. A rejection of this null leads to the determination that the predictive ability is greater than $\phi_{0}$, when these predictions are driven by a research hypothesis about the underlying natural phenomenon then this provides evidence in favor of the research hypothesis. To highlight these differences we empirically evaluated the power of both tests.

We simulated data with the following characteristics: we allowed n = (3, 10, 20, 50) per group and *m* = (6, 16, 26, 50). All samples are simulated from a multivariate Normal distribution; our proposed test does not require the data to be Normal, however, the OLS test does. Since the null hypothesis is different between the two tests the effect size for our proposed test is determined by the difference between the hypothesized $\phi_{0}$ which we set to 0.50 and the actual predictive ability which we simulated for values of 0.80 and 0.90. We examined four different traditional effect sizes, they are for group 2 relative to group 1:

1. δ=0.50: An increase of 0.50 standard deviations on all endpoints.
2. δ=±0.50: An increase of 0.50 standard deviations on half the endpoints, and a decrease of 0.50 standard deviations on the other half.
3. δ= Stagger: An increase of 0.5 m/M standard deviations for all M endpoints.^6^
4. δ=± Stagger: An effect size of 0.5 m/M standard deviations for all M endpoints, with the first half (the smallest ones) all negative and the largest half all positive.

These effect sizes allowed us to examine equidirectional effects (δ=0.50, δ= Stagger) (a motivating reason for the development of O'Brien's OLS test) as well as bidirectional effects (δ=±0.50, δ=± Stagger)). As an example, the ASL measures introduced in Section 1.1 were expected to increase (a positive outcome) after an intervention. An example where bidirectional effects would be incorporated together also comes from imaging. Diffusor tensor imaging can provide outcomes of mean diffusivity (MD) or fractional anisotropy (FA) across different regions of the brain, both measures provide information about the health of the brain and are typically collected and reported together. However, a decrease in MD would be an improvement, while an increase in FA would be an improvement.

For our proposed test the exact test was used for *m* ≤ 20. Covariance matrices were simulated in the same way as in the Normal approximation, with 50 covariance matrices simulated for each combination of n, *m* and δ, and 100 samples generated from each covariance matrix. Results for the power simulation are shown in Table 3.

**TABLE 3:** Empirical power estimates for our proposed test and O'Brien's OLS test

		n = 3			n = 10		
*m*	δ	OLS	$\phi_{0.80}$	$\phi_{0.90}$	OLS	$\phi_{0.80}$	$\phi_{0.90}$
6	0.50	0.118	0.099	0.167	0.832	0.210	0.362
	±0.50	0.031	0.107	0.165	0.050	0.220	0.367
	Stagger	0.065	0.053	0.090	0.478	0.097	0.175
	±Stagger	0.037	0.054	0.092	0.190	0.091	0.167
26	0.50	0.196	0.210	0.465	0.999	0.624	0.902
	±0.50	0.031	0.206	0.461	0.049	0.628	0.910
	Stagger	0.084	0.047	0.154	0.853	0.168	0.408
	±Stagger	0.047	0.047	0.015	0.368	0.165	0.410
50	0.50	0.269	0.303	0.704	1	0.850	0.989
	±0.50	0.031	0.310	0.696	0.051	0.855	0.990
	Stagger	0.122	0.043	0.210	0.977	0.226	0.611
	±Stagger	0.061	0.043	0.191	0.545	0.233	0.600
		n = 20			n = 50		
*m*	δ	OLS	$\phi_{0.80}$	$\phi_{0.90}$	OLS	$\phi_{0.80}$	$\phi_{0.90}$
6	0.50	0.95	0.30	0.52	0.999	0.392	0.631
	±0.50	0.05	0.316	0.517	0.211	0.379	0.635
	Stagger	0.777	0.151	0.255	0.954	0.231	0.379
	±Stagger	0.308	0.147	0.253	0.5217	0.226	0.270
26	0.50	1	0.842	0.985	1	0.942	0.999
	±0.50	0.055	0.835	0.987	0.061	0.997	1
	Stagger	0.988	0.320	0.626	1	0.581	0.894
	±Stagger	0.657	0.335	0.638	0.937	0.582	0.893
50	0.50	1	0.975	0.999	1	0.997	1
	±0.50	0.057	0.977	0.999	0.058	0.997	1
	Stagger	1	0.485	0.862	1	0.815	0.988
	±Stagger	0.845	0.483	0.871	0.994	0.820	0.991

Abbreviation: OLS, ordinary least squares.

The following conclusions can reasonably be made from the simulations.

1. The proposed test's power is not conditioned on whether the effects are equidirectional. When they are, the OLS test outperforms our proposed test unless the sample size is small. However, our test maintains good power with bidirectional effects, something the OLS test is not suited for.
2. The OLS test is powerful against the alternative hypothesis that every effect is in the same direction. While the OLS test is extremely insensitive (as would be expected) to effects that are in different directions.
3. For small samples sizes, n = 3 per group (N = 6 total sample), our proposed test outperforms the OLS test regardless of the effects examined, while after n = 10 the OLS test has greater power for equidirectional effects. However, it has been shown the OLS test is very liberal with small sample sizes due to the normal approximation, while we show in Table 4 that our test is not, thus making comparisons more difficult.
4. As n increases the power for both tests increases but much more so for the OLS test, while as *m* increases the power for both tests increase but much more so for our proposed test.

**TABLE 4:** Empirical Type I error estimates for our proposed test

	n = 3					n = 10			
δ	*m* = 6	*m* = 16	*m* = 26	*m* = 50	*m* = 6	*m* = 16	*m* = 26	*m* = 50
1	0.027	0.020	0.013	0.006	0.044	0.042	0.045	0.038	
0.50	0.010	0.003	0.002	0.0001	0.025	0.016	0.011	0.005	
1 (0.10)	0.005	0.0002	0.0	0.0	0.005	0.0004	0.0001	0.0	
Stagger	0.006	0.0006	0.0002	0.0	0.011	0.002	0.0008	0.0001	
		n = 20				n = 50			
1	0.047	0.052	0.048	0.049	0.051	0.050	0.048	0.042	
0.50	0.038	0.030	0.027	0.022	0.042	0.048	0.048	0.050	
1 (0.10)	0.006	0.0008	0.0004	0.0	0.011	0.002	0.0008	0.0	
Stagger	0.016	0.007	0.003	0.0004	0.029	0.014	0.008	0.003	

We also examined the Type I error control of our proposed method. We looked at (n = 3, 10, 20, 50) per group with *m* = (6, 16, 26, 50) for values of ϕ=0.50 against the null hypothesis of $\phi_{0}=0.50$. These were simulated with an effect size of 1, 0.5, a stagger, and with 1 endpoint of 1 and the rest with 0.1. All these effects were simulated as all positive, and with half negative, the results were the same for the Type I error as with the power simulation thus we present only the Type I error estimates with all positive endpoints. The covariance matrices were uniformly sampled from all positive definite covariance matrices. The results are shown in Table 4.

The simulations show that our proposed test maintains Type I error at or below the nominal level. Recall that the effect our test is trying to detect is the difference between ϕ and $\phi_{0}$. The ability to detect this is also somewhat dependent on the traditional effect size, that is, the standardized difference between two groups. Our simulation shows that when the effect size δ consists of very small effects, and when $\phi =\phi_{0}$ the probability of falsely rejecting the null hypothesis is very close to 0. When δ consists of larger effects, such as effect sizes of 1 for each endpoint, the Type I error is at the nominal 0.05 level. This is due to the fact that with small samples, and small effect sizes even if a prediction of the direction is correct, the actual median of the observed difference could be on the wrong side of 0 due to sampling error. When the predictive ability is the same as the null hypothesized ability this means that there is almost no chance of rejecting the null when it is false. It's also interesting to note that as *m* increases our Type I error decreases for fixed n and δ. Due to the fact that power also increases as *m* increases it is advantageous to incorporate as many endpoints that are related to the research hypothesis as possible. We also note here the difference between our proposed test and O'Brien's OLS test which has been shown to be liberal with small sample sizes.^10^ Conversely our test is conservative for small samples, for example, n = 3 in Table 4, while achieving greater power than O'Brien's OLS test for very small samples, n = 3 in Table 3, note that the n is per group. In general our proposed test is conservative when the effect sizes between groups are small, and achieves the nominal Type I error rate as the effect size increases.

### 6.2 ASL example

A dataset consisting of measurements of ASL in different regions of the brain will be used to demonstrate our test. This data comes from Dr. Vidoni at the University of Kansas Medical Center. The data was collected as part of a larger study examining the relationship between exercise and Alzheimer's disease that measured pre‐post changes after a 12 week exercise intervention in 11 older adults. The data we present here consists of ASL measures on six regions of the brain: BA46, Frontal mid, Hippocampus, M1, Superior Parietal and Precuneus, thus *m* = 6. The research hypothesis is that ASL will increase in the six regions of the brain following the intervention. No single region is of greater interest, and we take the observed result across these different regions as a proxy for the more general research hypothesis concerning structural/functional changes in the brain following the intervention. The researcher provided predictions were that blood flow would increase in every region. For this data we observed the following pre‐post difference sample means [0.16, 0.44, −1.49, 1.07, 0.97, −0.24] for BA46, Frontal mid, Hippocampus, M1, Superior Parietal and Precuneus, respectively. Thus, four of the six regions did actually increase meaning the predictions were correct on only two thirds of the measures. The results of the predictions are therefore **p** = [1, 1, 0, 1, 1, 0]^*T*^. For our dataset we calculated the differences in ASL in the six regions before and after the intervention and calculated the following sample correlation matrix of those differences.

We observed the following weights *w*_BA46_ = 0.46, *w*_FrontalMid_ = 0.41, *w*_Hippocampus_ = 0.68, *w*_M1_ = 0.48, *w*_Superior Parietal_ = 0.39 and *w*_Precuneus_ = 0.40, with $W=\sum_{i=1}^{6}w_{i}=2.22$.

Our observed test statistic is *t*_*m*_ = 1 · (0.46) + 1 · (0.41) + 0 · (0.68) + 1 · (0.48) + 1 · (0.39) + 0 · (0.40) = 1.74.

The weight given to the ASL measure in the Hippocampus is over 140% of the weight of any other measure, indicating that ASL measures in the Hippocampus were more “independent” of the other regions, thus the result of predictions in that region are given more weight.

There are 2^6^ possible combinations of predictions, thus our test statistic can take on 64 different values. The null hypothesis for this study was set at $\phi_{0}=0.50$. The probabilities of correctly predicting {0, 1, 2, 3, 4, 5, 6} responses are (0.015625, 0.09375, 0.234375, 0.3125, 234375, 0.09375, 0.015625), these are simply binomial probabilities with success parameter 0.50 and n = 6. There are (1, 6, 15, 20, 15, 6, 1) combinations, respectively, of getting the {0, 1, 2, 3, 4, 5, 6} correct predictions. Thus, the probability of any single test statistic value depends on the number of correct predictions shown below.

$$\begin{array}{ll} & \left[\frac{0.015625}{1},\frac{0.09375}{6},\frac{0.234375}{15},\frac{0.3125}{20},\frac{0.234375}{15},\frac{0.09375}{6},\frac{0.015625}{1}\right]\;\\ & \;=\left[0.015625,0.015625,0.015625,0.015625,0.015625,0.015625\right].\; \end{array}$$

In the case where $\phi_{0}=0.5$ the probability of any single test statistic value will be the same as any other value, however, this is not the case when $\phi_{0}\neq 0.5$. Given the probabilities of all possible test statistic values, we can then calculate the distribution of the test statistic exactly since we can enumerate the probability of every possible value and could calculate every value.

For our observed *t*_*m*_ = 1.74 with $\phi_{0}=0.50$ the probability of getting a test statistic as or more extreme under the null, that is, the *p*‐value, is .3125, thus we would fail to reject the null hypothesis at the traditional α=0.05 level in favor of the alternative that the research's true predictive ability is ≥ 0.50. We note that if t‐tests had been carried out on all 6 measures, and one specified as primary, with the others as secondary, it would not matter which one was designated as primary because the *p*‐value for all six tests is >.05. This dataset also could be analyzed using O'Brien's OLS test since the predicted response was in the same direction for each endpoint. The OLS test resulted in a test statistic of *T*_OLS_ = 0.03, with *p*‐value of .86. Both our proposed test and O'Briens OLS test came to similar conclusions. The interpretation for the OLS test is that there is little evidence that the effect of the intervention was positive across all endpoints, namely, because they pre‐post changes were not all positive. Our proposed test can be interpreted as providing little evidence that the researcher was able to predict the directional changes, indicating that the research hypothesis which informed those predictions was not supported by the data.

## 7 DISCUSSION

We have described a new statistical test for studies with multiple endpoints. Our technique makes use of researcher's predictions and is essentially a test of whether a researcher's understanding of the natural process, that is, their ability to correctly predict the outcomes, is convincing enough to support their research hypothesis. This test is able to answer an essential question in early biomedical research, does the researcher understand enough about the natural process to continue supporting that research, or should it be abandoned in favor of a more promising research track. Our test has an exact distribution as well as a normal approximation for use when the computation of the exact distribution becomes difficult. We compared our test to O'Brien's OLS test which has been used extensively in settings with multiple endpoints and found several key differences. O'Brien's OLS test is more powerful when the effects on each endpoint are all in the same direction except when the sample size is very small. While this may sometimes be the case, it is certainly a restriction on the applicability of the test to situations where some of the endpoints may be expected to change in different directions. Our proposed test uses all endpoints to come to a single conclusion about a research hypothesis but does not restrict the directional change to one direction. We have shown that our test performs well in those situations and when all effects are in the same direction for a large number of endpoints. Importantly, our test has an exact distribution and can be used for extremely small samples (simulation showed N = 6, with 3 per group) and many endpoints, situations under which similar tests that rely on approximations, such as the OLS test or Hotelling's *T*^2^ are unreliable.

### 7.1 Limitations, extensions, and future work

For small sample sizes it has been shown that sample correlations can be unstable.^18^, ^19^ This is the main concern with treating the weights as fixed since for large sample sizes the sample pairwise correlations and thus the weights will be close to their true values. The literature on the topic has been concerned with the stability of estimates for hypothesis testing while we are interested in the point estimate; nevertheless, for small sample sizes the instability of the pairwise sample correlations could lead to our weights being far from the true value and our test might over or under weight various endpoints. While this is a concern, we note that our test came to the same conclusions over 96% of the time in all scenarios we examined in Section 6.

We believe there are many ways to extend this methodology. In this article, we have only included one‐sided predictions, however, predictions of “change” or “no‐change” can also be made. For example, with a prediction of a “change” that is a difference from 0 by making an assumption about the distribution, such as Normal, of the endpoint we can define an interval around 0 such that there is $\phi_{0}$ probability of an observation being within that interval. An observed value outside the interval would be considered a correct prediction.

One assumption of our approach is that the predictive ability on all endpoints is constant, this may not be realistic in scenarios where the researcher has better information about one endpoint relative to another. For instance, a researcher could be very certain that endpoint A would increase and set $\phi_{0,A}=0.8$ while they might be less certain that endpoint B would have their predicted change and set $\phi_{0,B}=0.5$. If the predictive ability differed across endpoints both the mean and variance of our proposed test statistic would change. Under this null distribution the sum of the responses from these endpoints could be modeled as a weighted Poisson Binomial Distribution which can be calculated via a recursive formula for small samples^20^ or can be approximated with larger samples.

Our current work and plans for future work include: extending the methodology beyond one sided predictions to encompass the two sided predictions discussed and allowing different prediction probabilities for different endpoints, thereby assigning more importance to different endpoints. In addition, further work needs to be done to compare our test to other nonparametric tests such as O'Brien's RS test under various settings.

## Data Availability

The data used in the example for this study are available from the corresponding author upon reasonable request.

## APPENDIX A ASYMPTOTIC NORMAL APPROXIMATION

### A.1

The Lindberg^14^ Condition states that for a set of independent but not necessarily identically distributed random variables *X*_*i*_ with expected values $\mu_{i}$ and variances $\sigma_{i}^{2}$ where we let $s_{m}^{2}=\sum_{i=1}^{m}\sigma_{i}^{2}$ that

$$\overset{m}{\underset{i=1}{\sum }}\frac{X_{i}-\mu_{i}}{\sqrt{\sum_{i=1}^{m}\sigma_{i}^{2}}}\overset{d}{\underset{}{\to }}N(0,1)$$

as long as the following condition holds for all ϵ>0

$$\underset{m\to \infty }{\lim}\frac{1}{s_{m}^{2}}\overset{m}{\underset{i=1}{\sum }}E\left[{\left(X_{i}-\mu_{i}\right)}^{2}\cdot 1_{|X_{i}-\mu_{i}|>\epsilon \cdot s_{m}}\right]=0$$

We show that as long as $\phi_{0}$ is bounded away from 0 and 1 this condition will hold. We consider the approximation for $\phi_{0}\in (0,1)$ and we do not consider the cases where *C* = *I* or *C* = *J*_*m*_ since we have shown that in both cases the test statistic follows a binomial distribution and thus has a well‐known normal approximation. We also make the assumption that as *m* → *∞* the number of unique $w_{i}^{\prime }s$, the weights, also goes to infinity. By assuming this we restrict this approximation to settings where the measure of interest are not all equal, or for instance, where all but 1, or 2 are all equal. We assume here that the number of unique measures grows.

Let *X*_*i*_ = *p*_*i*_*w*_*i*_ then under the null hypothesis and treating the weights as fixed leads to $E[X_{i}]=\phi_{0}w_{i}$ and $\sigma_{X_{i}}^{2}=\phi_{0}(1-\phi_{0})w_{i}^{2}$ and $s_{n}=\sqrt{\phi_{0}(1-\phi_{0})\sum_{i=1}^{m}w_{i}^{2}}$ gives the following formulation of the Lindberg condition

$$\underset{m\to \infty }{\lim}\frac{1}{\phi_{0}(1-\phi_{0})\sum_{i=1}^{m}w_{i}^{2}}\overset{m}{\underset{i=1}{\sum }}E\left[{\left(p_{i}w_{i}-\phi_{0}w_{i}\right)}^{2}\cdot 1_{|p_{i}w_{i}-\phi_{0}w_{i}|>\epsilon \cdot \sqrt{\phi_{0}(1-\phi_{0})\overset{m}{\underset{i=1}{\sum }}w_{i}^{2}}}\right]=0$$

The LHS of the limit $\left(\frac{1}{\phi_{0}(1-\phi_{0})\sum_{i=1}^{m}w_{i}^{2}}\right)$ will always go to 0 as *m* → *∞* because since *w*_*i*_ > 0 for all *i* and we assume the weights are not all identical the sum of the squared weights will go to *∞* as *m* → *∞*.

Thus, we focus on the expectation and specifically on the indicator function. We show that for given restriction of $\phi_{0}$ the indicator will always be 0, thus the expectation will always be 0 and thus the limit as *m* → *∞* equals 0, satisfying the condition and we show that these restrictions on $\phi_{0}$ will always be satisfied as *m* → *∞*.

There are two scenarios to consider the indicator function under, when *p*_*i*_ = 0 and when *p*_*i*_ = 1.

**For all i such that** *p*_*i*_ **=** **0 :**

Note that *w*_*i*_ and $\phi_{0}$ are always > 0. The indicator function depends on

$$\begin{array}{ll} \left|-\phi_{0}w_{i}\right| & >\epsilon \sqrt{\phi_{0}(1-\phi_{0})\overset{m}{\underset{i=1}{\sum }}w_{i}^{2}}\;\\ =\phi_{0}^{2}w_{i}^{2} & >\epsilon^{2}\phi_{0}(1-\phi_{0})\overset{m}{\underset{i=1}{\sum }}w_{i}^{2}\;\\ =\phi_{0}w_{i}^{2} & >\epsilon^{2}(1-\phi_{0})\overset{m}{\underset{i=1}{\sum }}w_{i}^{2}\;\\ =\phi_{0}w_{i}^{2} & >\epsilon^{2}\overset{m}{\underset{i=1}{\sum }}w_{i}^{2}-\phi_{0}\epsilon^{2}\overset{m}{\underset{i=1}{\sum }}w_{i}^{2}\;\\ -\phi_{0}(w_{i}^{2}+\epsilon^{2}\overset{m}{\underset{i=1}{\sum }}w_{i}^{2}) & >\epsilon^{2}\overset{m}{\underset{i=1}{\sum }}w_{i}^{2}\;\text{(combining like terms)}\;\\ \;\Rightarrow \;\phi_{0} & >\frac{\epsilon^{2}\sum_{i=1}^{m}w_{i}^{2}}{\left(w_{i}^{2}+\epsilon^{2}\sum_{i=1}^{m}w_{i}^{2}\right)}.\; \end{array}$$

Thus, when *p*_*i*_ = 0 the indicator function will be 1 for all *i* if the above inequality holds, thus the indicator function will be 0 for all *i* such that *p*_*i*_ = 1 if the following inequality holds.

$$\begin{array}{ll} \phi_{0} & <\frac{\epsilon^{2}\sum_{i=1}^{m}w_{i}^{2}}{\left(w_{i}^{2}+\epsilon^{2}\sum_{i=1}^{m}w_{i}^{2}\right)}.\; \end{array}$$

**For all i such that** *p*_*i*_ **=** **1 :** Note that *w*_*i*_ and $\phi_{0}$ are always > 0. The indicator function depends on

$$\begin{array}{ll} \left|w_{i}-\phi_{0}w_{i}\right| & >\epsilon \sqrt{\phi_{0}(1-\phi_{0})\overset{m}{\underset{i=1}{\sum }}w_{i}^{2}}\;\\ \left|w_{i}(1-\phi_{0})\right| & >\epsilon \sqrt{\phi_{0}(1-\phi_{0})\overset{m}{\underset{i=1}{\sum }}w_{i}^{2}}\;\\ w_{i}^{2}{\left(1-\phi_{0}\right)}^{2} & >\epsilon^{2}\phi_{0}(1-\phi_{0})\overset{m}{\underset{i=1}{\sum }}w_{i}^{2}\;(\text{removing absolute value and squaring})\;\\ w_{i}^{2}(1-\phi_{0}) & >\epsilon^{2}\phi_{0}\overset{m}{\underset{i=1}{\sum }}w_{i}^{2}\;\\ w_{i}^{2}-w_{i}^{2}\phi_{0} & >\epsilon^{2}\phi_{0}\overset{m}{\underset{i=1}{\sum }}w_{i}^{2}\;\\ w_{i}^{2} & >\left(\epsilon^{2}\overset{m}{\underset{i=1}{\sum }}w_{i}^{2}+w_{i}^{2}\right)\phi_{0}\;\\ \frac{w_{i}^{2}}{\epsilon^{2}\sum_{i=1}^{m}w_{i}^{2}+w_{i}^{2}} & >\phi_{0}.\; \end{array}$$

Thus, when *p*_*i*_ = 1 the indicator function will be 1 for all *i* if the above inequality holds, therefore the indicator function will equal 0 for all *i* such that *p*_*i*_ = 1 if the following inequality holds.

$$\frac{w_{i}^{2}}{\epsilon^{2}\sum_{i=1}^{m}w_{i}^{2}+w_{i}^{2}}<\phi_{0}$$

These two scenarios, that for a given *i* *p*_*i*_ = 0 or *p*_*i*_ = 1 cover all possible outcomes. When we combine these two restrictions on $\phi_{0}$ they give an upper and lower bound for $\phi_{0}$ such that the indicator function will equal 0 if for all *i* $\phi_{0}$ is within

$$\left(\frac{w_{i}^{2}}{\epsilon^{2}\sum_{i=1}^{m}w_{i}^{2}+w_{i}^{2}},\frac{\epsilon^{2}\sum_{i=1}^{m}w_{i}^{2}}{(w_{i}^{2}+\epsilon^{2}\sum_{i=1}^{m}w_{i}^{2}}\right)$$

If this holds then the indicator function will always be 0 and the limit will equal 0, thus the Lindberg condition will hold. We note that as *m* → *∞* the summation $\sum_{i=1}^{m}w_{i}^{2}$ will tend to infinity so long that as *m* increases the number of unique weights also increases (ie, the increase in *m* is not solely driven by adding copies of the same measure). Thus, as the lower bound will tend to 0, and the upper bound will tend to 1, so the restriction on $\phi_{0}$ tends to (0, 1) as *m* → *∞*. Thus, asymptotically the normal approximation will hold so long as $\phi_{0}$ is not set equal to 0, or 1.
